# Potent antitumor efficacy of human dental pulp stem cells armed with YSCH-01 oncolytic adenovirus

**DOI:** 10.1186/s12967-023-04539-z

**Published:** 2023-10-03

**Authors:** Xu He, Wei Yao, Ji-Ding Zhu, Xin Jin, Xin-Yuan Liu, Kang-Jian Zhang, Shou-Liang Zhao

**Affiliations:** 1 Department of Stomatology, Huashan Hospital, Fudan University, 12 Urumqi Road, Jing’an District, Shanghai, 200040 China; 2 Academician Expert Workstation of Fengxian District, Shanghai Yuansong Biotechnology Limited Company, 1588 Huhang Road, Fengxian District, Shanghai, 201499 China; 3 Institute of Smart Biomedical Materials, School of Materials Science and Engineering, Zhejiang Sci-Tech University, 928 Second Avenue, Xiasha Higher Education Zone, Hangzhou, 310018 China; 4 State Key Laboratory of Cell Biology, Shanghai Institute of Biochemistry and Cell Biology, Center for Excellence in Molecular Cell Science, Chinese Academy of Sciences, 320 Yueyang Road, Xuhui District, Shanghai, 200031 China; 5 Shanghai Fengxian Stomatological Hospital, 189 Wanghe Road, Fengxian District, Shanghai, 201499 China; 6 Department of Stomatology, School of Medicine, Renji Hospital, Shanghai Jiaotong University, 160 Pujian Road, Pudong New Area, Shanghai, 200025 China

**Keywords:** Human dental pulp stem cell, Oncolytic adenovirus, Cell carrier, Tumor tropism, Intraperitoneal injection, Cancer therapy

## Abstract

**Background:**

Systemic administration of oncolytic adenovirus for cancer therapy is still a challenge. Mesenchymal stem cells as cell carriers have gained increasing attention in drug delivery due to their excellent tumor tropism, immunosuppressive modulatory effects, and paracrine effects. However, the potential of human dental pulp stem cells (hDPSCs) loaded with oncolytic adenovirus for cancer biotherapy has not been investigated yet.

**Methods:**

The stemness of hDPSCs was characterized by FACS analysis and Alizarin red staining, Oil Red O staining, and immunofluorescence assays. The biological fitness of hDPSCs loaded with oncolytic adenovirus YSCH-01 was confirmed by virus infection with different dosages and cell viability CCK-8 assays. Additionally, the expression of CAR receptor in hDPSCs was detected by qPCR assay. Tumor tropism of hDPSC loaded with YSCH-01 in vitro and in vivo was investigated by Transwell assays and living tumor-bearing mice imaging technology and immunohistochemistry, Panoramic scanning of frozen section slices assay analysis. Furthermore, the antitumor efficacy was observed through the different routes of YSCH-01/hPDSCs administration in SW780 and SCC152 xenograft models. The direct tumor cell-killing effect of YSCH-01/hDPSCs in the co-culture system was studied, and the supernatant of YSCH-01/hDPSCs inhibited cell growth was further analyzed by CCK-8 assays.

**Results:**

hDPSCs were found to be susceptible to infection by a novel oncolytic adenovirus named YSCH-01 and were capable of transporting this virus to tumor sites at 1000 VP/cell infectious dosage in vitro and in vivo. Moreover, it was discovered that intraperitoneal injection of hDPSCs loaded with oncolytic adenovirus YSCH-01 exhibited potential anti-tumor effects in both SW780 and SCC152 xenograft models. The crucial role played by the supernatant secretome derived from hDPSCs loaded with YSCH-01 significantly exerted a specific anti-tumor effect without toxicity for normal cells, in both an active oncolytic virus and an exogenous protein-independent manner. Furthermore, the use of hDPSCs as a cell carrier significantly reduced the required dosage of virus delivery in vivo compared to other methods.

**Conclusions:**

These findings highlight the promising clinical potential of hDPSCs as a novel cell carrier in the field of oncolytic virus-based anti-cancer therapy.

## Background

Oncolytic adenovirus is extensively studied for clinical cancer therapy [1, 2] due to its promising anti-tumor effect and reduced toxicity to normal tissues [3, 4]. However, challenges such as the presence of neutralizing antibodies, complement, anti-viral immunity, and limited tumor tropism hinder the effective systemic administration of oncolytic adenovirus. Consequently, novel methods are being urgently explored to overcome these obstacles. Intratumoral administration remains the most effective approach for oncolytic adenovirus therapy to date, considering the existence of neutralizing antibodies, complement, anti-viral immunity, and weak tumor tropism. Nonetheless, researchers are actively investigating alternative strategies to enable its systemic administration. These approaches can be broadly categorized into three typical strategies: the exploration of next-generation oncolytic viruses [5], combination with intelligent biomaterials [6, 7], and utilization of cell carriers [8, 9].

Cell carriers offer several advantages by facilitating the escape of loaded viruses from host anti-virus immunity and improving their efficient targeting of distant tumor sites. Various types of cell carriers have been identified, including cells with lymphocyte characteristics such as cytokine-induced killers, peripheral blood lymphocytes, dendritic cells, and cells with tumor cell properties such as tumor-associated macrophages. Additionally, cells with stemness, primarily mesenchymal stem cells (MSCs) and endothelial progenitor cells, have demonstrated potential as a cell carrier [10, 11].

MSCs have gained increasing attention in drug delivery due to their excellent tumor tropism, immunosuppressive modulatory effects, and paracrine effects. Studies have reported significant inhibition of tumor growth and reduced hepatotoxicity following intravenous injection of bone marrow-derived MSCs loaded with oncolytic virus HCC-oAd [12]. Moreover, MSCs have shown the ability to be infected by oncolytic herpes simplex virus (HSV) and exhibited anti-tumor efficacy in a preclinical model of malignant glioblastoma multiforme [13]. These findings suggest that combining MSCs with oncolytic viruses holds promise as a strategy for systemic administration.

Odontogenic stem cells, a type of MSC, are primarily utilized in tissue regeneration due to their ease of acquisition and low immunogenicity. Human dental pulp stem cells (hDPSCs) exhibit either very low or no expression of MHC-II molecules and limited expression of MHC-I, rendering them less recognizable by recipient T cells to escape immune rejection reaction [14]. Phase II clinical trials with dental pulp stem cells have been initiated in China (NCT05924373). Besides that, hDPSCs have been reported to possess the ability to migrate to inflammatory sites facilitated by their membrane receptors and the release of chemokines, inflammatory factors, cytokines, and immune factors. Among the most well-known signaling pathway is the CXCR4/SDF-1α axis [15]. However, a study investigating subcutaneous, intravenous, and peritumoral injection of hDPSCs into subcutaneous tongue cancer models revealed no evidence of stem cell presence in the tumor tissue [16]. This observation suggests that hDPSCs may not effectively migrate into tumors. Unfortunately, no other relevant reports on hDPSCs’ tumor tropism and their potential application as a cell carrier for delivering oncolytic adenovirus were found.

In this study, a novel oncolytic adenovirus named YSCH-01(ClinicalTrials.gov Identifier: NCT05180851) was selected. For the first time, hDPSCs loaded with YSCH-01 demonstrated tumor tropism and exhibited a potent antitumor secretome derived from YSCH-01/hDPSCs. These findings highlight the clinical potential of hDPSCs as a novel cell carrier in the field of oncolytic adenovirus therapy.

## Methods and materials

### Culture and identification of human dental pulp stem cells

Human dental pulp stem cells (hDPSCs) were obtained from Shanghai Fengxian Stomatological Hospital and cultured following the previously described [17]. In brief, the cells were cultured in α-MEM medium supplemented with 10% fetal bovine serum (FBS; Sciencell) and 1% penicillin–streptomycin (Sigma). The preferential hDPSCs were selected using a fibronectin adhesion assay based on their high expression of β1-integrin [18]. For this study, hDPSCs at passages 3–9 were used. The selected cells were confirmed to express the cell surface makers CD44, CD90, and Stro-1 through flow cytometry analysis. The multilineage differentiation ability of hDPSCs was evaluated using Alizarin red staining, Oil Red O staining, and immunofluorescence, following induction with osteogenic media for 14 days, adipogenic media for 28 days, and neural induction media for 8 days, according to the previous protocol [19–21].

### Oncolytic virus

The oncolytic adenovirus utilized in this study, named YSCH-01, was developed by Shanghai Yuansong Biotechnology Co., Ltd. And has progressed to the clinical research stage (ClinicalTrials.gov Identifier: NCT05180851). YSCH-01 is a double-regulated replicative human adenovirus type 5 vector carrying an optimized recombinant interferon-like anti-tumor gene. The control virus, OncoMul-V2-EGFP, armed with the EGFP gene, shares the same viral backbone as YSCH-01. All the viruses were stored at −80 °C, and the freeze–thaw cycles did not exceed three times.

### Construction of hDPSCs loaded with oncolytic adenovirus

**Fig. 1:**
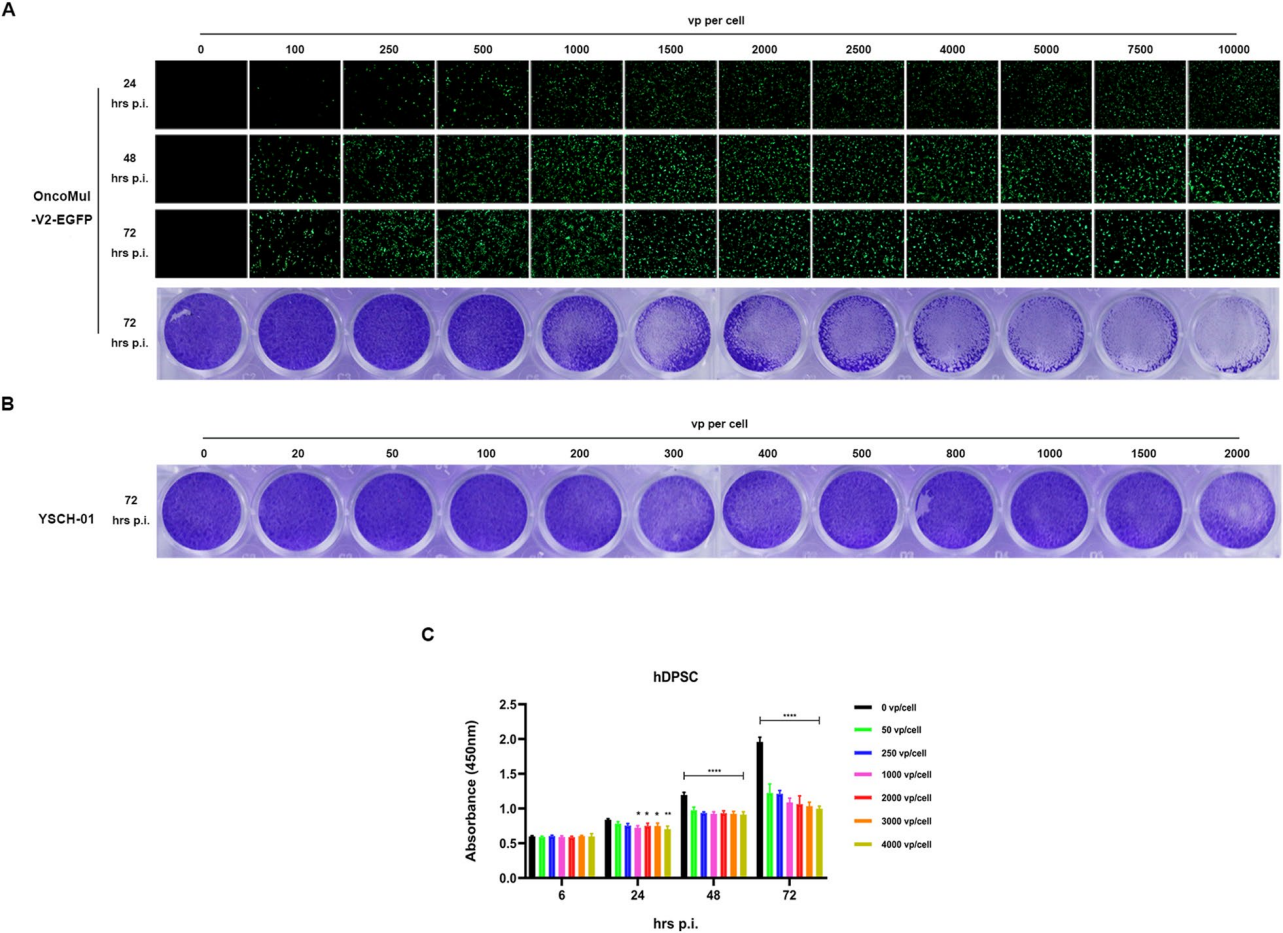
Characterization of hDPSCs loaded with oncolytic adenovirus. **A** EGFP expression level in hDPSCs infected with OncoMul-V2-EGFP and the magnification is 40. The bottom panel is the Crystal violet staining photos of hDPSCs treated with increased dosages of OncoMul-V2-EGFP for 72 h. **B** The Crystal violet staining results of hDPSCs infected by oncolytic adenovirus YSCH-01 at different infectious doses. **C** The cell viability of hDPSCs treated with different infectious doses of YSCH-01 at the indicated time points

hDPSCs were plated at a density of 4 × 10^4^ cells/cm^2^. After 16 h of plating, freshly thawed virus stock was added to the culture medium at different indicated infective doses, mixed, and incubated at 37 °C in a 5% CO2 environment for 4 h. The cells were then washed twice with PBS and subjected to enzymatic digestion to obtain virus/hDPSC complexes. The fluorescence images in Fig. 1 were obtained at 24 h, 48 h, and 72 h post-infection, while the images in Appendix Fig. 7B were observed after virus infection for 4 h, 7 h, and 12 h, followed by a 72-h culture. In the context of a 4-h, 7-h, and 12-h virus infection, it means that after being infected with the virus for 4 h, 7 h, or 12 h, the supernatant was discarded and replaced with fresh culture medium. For subsequent experiments related to anticancer drug efficacy and mechanisms, YSCH-01/hDPSCs were infected at a dose of 1000 VP/cell for 4 h. The virus/hDPSC complexes, including YSCH-01/hDPSC, were used immediately after infection in each experiment and were not subjected to freezing.

### Cell lines

The human tumor cell lines used in this study include the SCC152 glossopharyngeal cancer cell line (ATCC CRL-3240), SW780 bladder cancer cell line (ATCC CRL-2169), and HCC1806 breast squamous cancer cell line (ATCC CRL-2335). Additionally, an interferon receptor signaling deficiency (IFNAR2^−/−^) U5A fibrosarcoma cell line, generously provided by Prof. Zhengfan Jiang (Peking university), was used. SCC152, HCC1806, and U5A were cultured in DMEM (Dulbecco's modified eagle’s medium, POWER CELL PWL003), while SW780 were cultured in RPMI 1640 medium (Gibco C22400500BT), both supplemented with 10% FBS. The 293A cell line (ThermoFisher, R70507) was cultured in DMED with 10% FBS. MCF-10A normal breast epithelial cells were cultured in MEBM (Lonza, CC-3151) with MEGM SingleQuots (Lonza, CC-4136). All the aforementioned cells were incubated in a 37℃, 5% CO_2_ incubator. SCC152-EGFP and SW780-EGFP cells, as well as hDPSC-RFP, were generated using Lentivirus technology as previously described [22].

### Crystal violet staining

Following fixation, the cells were exposed to 2% crystal violet solution for 15 min. Subsequently, the cells were washed with running water and photographed.

### Colony and cell sphere formation

The self-renewal ability of hDPSC was identified with a colony and cell sphere formation. As for the colony formation, the experiment used a six-well plate for colony formation, with 500 hDPSCs seeded in each well. After 8 days, cell morphology images were studied under a microscope, and colony formation was stained with 2% crystal violet dye. In the sphere formation experiment with hDPSCs, 2500 cells were seeded in an ultra-low-attachment 6-well plate in 2 ml culture medium. Fresh hDPSC culture medium was supplemented by 1 ml every 2 days, and after 8 days, photographs were taken to observe the formation of spheres.

### Cell counting kit-8 (CCK8) assay

Cell proliferation was assessed using the Cell Counting Kit-8 (Dojindo, Kumamoto, Japan). HDPSCs were seeded in a 96-well plate at a density of 5000 cells/100 μl per well and incubated in a humidified incubator at 37 ℃, 5% CO_2_ for 24 h. The cells were then treated with different dosages of YSCH-01 viruses. After 72 h, 10 μl of CCK8 reagent was added to each well and further incubated at 37 ℃, 5% CO_2_ for 3 h. Finally, the absorbance at 450 nm was measured using a microplate reader (Thermofisher Scientific, Multiskan FC).

### Transwell assay

For the transwell assay, 1.5 × 10^6^ tumor cells were seeded in the lower chamber and allowed to adhere for 24 h. Subsequently, 2 × 10^4^ YSCH-01/hDPSC complexes were added to the upper chamber. After co-culturing for 16 h, the hDPSCs were fixed with 4% paraformaldehyde for 30 min and stained with 2% crystal violet solution. Microscopic images were captured from five fields, including the upper, lower, left, right, and middle areas of each well. Statistical analysis was performed based on the acquired images.

### Anti-tumor effect on xenograft models

All animal experiments were conducted in accordance with the guidelines and approved by the institutional Ethics Committee (IACUC), Shanghai LideBiotech Co., Ltd (LDIACUC 006). Data analysis was performed in a single-masked manner. Two investigators were assigned specific responsibilities for each animal. One investigator was responsible for model establishment, groups allocation, and treatment, while the other was in charge of data acquisition and assessment. Animals with missing labels were excluded from the study, and no animals were excluded in these experiments. Female Balb/c nude mice aged 6–8 weeks were obtained from Shanghai Lingchang Biotechnology Co., Ltd. The mice were used to establish subcutaneous SW780 bladder and SCC152 glossopharyngeal tumor-bearing models. For the SW780 xenograft model, a total of 25 mice were randomly divided into 5 groups: Mock group (n = 5), hDPSC-IP group (n = 5), YSCH-01/hDPSC-IP group (n = 5), hDPSC-IV group (n = 5), and YSCH-01/hDPSC-IV group (n = 5). In the SCC152 xenograft model, a total of 20 mice were randomly divided into 4 groups: Mock group (n = 5), YSCH-01/hDPSC-IV group (n = 5), YSCH-01/hDPSC-IP group (n = 5), and YSCH01-IT group (n = 5). Administration of treatment started when the corresponding tumors reached a size of 80–100 mm^3^. Each injection dose in YSCH-01-IT group was 2 × 10^9^ VP, and each injection of hDPSC or YSCH-01/hDPSC consisted of 2 × 10^6^ cells or 2 × 10^6^ YSCH-01/hDPSC complexes, with an infection dosage of 1000 VP/cell and infection for 4 h. And in the experiment as shown in Appendix Fig. 8, the low (2 × 10^5^ VP), medium (2 × 10^6^ VP), and high doses (2 × 10^7^ VP) of YSCH-01 were injected intraperitoneally at each injection. The injections were administered once every four days, for a total of 5 injections, via intraperitoneal, intravenous, or intratumor routes, depending on the assigned group. Tumor size and mouse body weight were measured every 3 days. The calculation formula for tumor size (mm^3^) was as follows: length (mm) × width ^2^ (mm^2^)/2. The sample size for all animal experiments was predetermined based on calculations derived from our laboratory experience. To minimize potential confounders, the age and sex of nude mice, the order of treatments and measurements, and the location of animal cages were standardized.

### Immunohistochemistry

The standard immunoperoxidase staining procedure for immunohistochemistry was performed using a previously described protocol [23]. Briefly, tumors were harvested from xenograft mice on day 4 post-treatment and fixed in 4% paraformaldehyde. The fixed tumors were then embedded in paraffin and cut into 8 μm sections. The tumor sections were stained using a primary mouse anti-adenoviral hexon antibody (Novus, NB600-413) at a 1:100 dilution. After staining, the slides were washed with 1 × PBS buffer, and the following steps for secondary antibody staining using a GOAT anti-mouse secondary antibody were performed according to the kit instruction of the avidin–biotin-peroxidase complex reagent (Vector Laboratories, Burlingame, CA). The immunohistochemistry sections were analyzed using KFBIO KF-PRO-120 digital pathology slide scanner.

As for the identification of neural-like cell, Immunocytochemical analysis was carried out for the detection of cell-specific markers [24]. Briefly, fixed cells were blocked with phosphate-buffered saline containing 10% normal rabbit serum (Sera Laboratories International Ltd) and 0.3% Triton-X 100, followed by overnight incubation in antibodies at 4 ℃. The primary antibodies were mouse anti-human Nestin (1:200 dilution, 66259-1-Ig, ProteinTech) and rabbit anti-human GFAP (1:300, Ab68428, Abcam). Secondary antibodies were Cy3-conjugated rabbit anti-mouse IgG (1:250; Runnerbio) and Cy5-conjugated goat anti-rabbit IgG (1:250; Runnerbio). Fluorescent images were captured from the counterstained slides using a Nikon DS-Fi2 microscope (Nikon) and Image-Pro AMS version 6.0 software.

### Viral titer assay

Stem cells were plated at a density of 2 × 10^5^ cells per well in a 6-well plate and infected with YSCH-01 as described above. The YSCH-01/hDPSC complexes were washed with PBS three times, and the cellular lysis of the YSCH-01/hDPSC complex was obtained by freeze-thawing three times at – 80 ℃. To further detect the titer of intracellular adenovirus, an immunocytochemical staining assay was performed according to the kit instructions (Cell Biolabs, VPK-109). Briefly, 293A cells were plated in a 24-well plate at a density of 2.5 × 10^5^ cells, and 100ul of YSCH-01/hDPSC cellular lysis was added to the 293A cells overnight. After 48 h of infection, the cells were fixed with pre-cooled methanol for 20 min and washed three times with 1 × PBS buffer. The cells were then blocked with 1% BSA for 1 h, and the adenovirus hexon antibody, diluted 500-fold, was added. After incubation at room temperature for 1 h, the cells were washed 3 times with PBS. Next, a secondary antibody, diluted 1000-fold, was added to the 293A cells and incubated at 37 ℃ for 2 h, followed by washing with PBS buffer three times. Then, a DAB staining solution was added and incubated for 20 min. Finally, nine fields of each well were photographed under a 10 × objective lens, and the average counting numbers of positively stained particles were calculated. The virus titer (ifu/ml) formula described in the kit was used to calculate the titer, with the sole YSCH-01 group serving as the positive control.

### Co-culture assay

Passage 6 of hDPSC-RFP, SCC-152-EGFP, and SW780-EGFP stable cell lines were constructed using lentivirus transfection and selection technology [22]. The transfected tumor cells and hDPSCs were pre-cultured in separate 6-well plates and 6-cm dishes, respectively, before the co-culture system. The hDPSC-RFP cells were infected with the YSCH-01 virus at a dose of 1000 VP/cell for 4 h, as described above. Then, the YSCH-01/hDPSC-RFP cells were trypsinized and diluted in an FBS-free medium. Co-cultured YSCH-01/hDPSC-RFP cells, as effector (E), were added to wells seeded with SCC152-EGFP, SW780-EGFP targeting (T) cells, in 6-well plates at effector-to-target ratios of 1:1, 1:2, and 1:5 respectively. The cell morphology in the co-culture system was documented by taking photos using an inverted fluorescence microscope at different time points. The untreated cells served as control groups.

### Cell tracking assays

The biodistribution of hDPSCs or YSCH-01/hDPSCs after systemic administration was detected using the Living Image of Animal Assay and Fluorescence Protein Labeling Assay. SW780 xenograft nude mice models were constructed as described above. The groups included Mock (n = 3), hDPSC-IV (n = 3), hDPSC-IP (n = 3), and YSCH-01/hDPSC-IP (n = 3). Briefly, hDPSCs or YSCH-01/hDPSCs were stained with fluorescent dye DiIC18(7)1,1’-dioctadecyltetramethyl indotricarbocyanine Iodide (DIR, Fluorecence Bio, 22070) for 30 min and injected into nude mice via intraperitoneal and intravenous administration, respectively. Living images were taken at different time points post-injection using the VISQUE invivo Smart-LF Spectrum to monitor cell distribution in vivo. The fluorescence radiant efficiency was also analyzed. Similarly, the groups were divided into hDPSC-IP (n = 3) and YSCH-01/hDPSC-IP (n = 3). Fluorescence protein-labeled cells, such as hDPSC-RFP, YSCH-01/hDPSC-RFP, and SW780-EGFP, were constructed as previously mentioned. Ten days after intraperitoneal injection of red-labeled hDPSCs or YSCH-01/hDPSCs, tumors were isolated and embedded in optimum cutting temperature compound buffer (SAKURA, catalog number 6502) for further frozen section analysis [25]. Briefly, 8 μm frozen tumor tissue sections were prepared using a cryotome, stained with DAPI, and observed for both red and green fluorescence protein distribution at the tumor sites using a digital slice scanner (3DHISTECH, panoramic MIDI) to demonstrate the homing of stem cells to the tumor in vivo.

### Assays on neutralization antibody of adenovirus

The neutralization antibody of YSCH-01 was obtained using the method described by Chen et al. [6]. Briefly, Balb/c mice were intravenously injected with 1.5 × 10^10^ YSCH-01 VP on the 1st and 14th day. After 14 days from the second injection, the anti-Ad5 antibody serum was separated and collected from the mice blood. The collected serum was stored at – 80 ℃. Different dilutions of the anti-Ad5 serum were mixed with the supernatant of YSCH-01/hDPSCs and incubated for one hour at 37 ℃. The mixture was then added to culture the SW780-EGFP cells instead of the normal medium. After 72 h, the cell morphology was observed, and the cell viability was measured using the CCK8 assay.

### Obtention of supernatant and its cellular killing assay

Passage 6 hDPSCs were cultured at a density of 4.5 × 10^6^ in a T175 Flask. After 18 h, the adhered cells were infected with Oncolytic adenovirus YSCH-01 at a dosage of 1000 VP per cell, while the control group was treated with the virus buffer alone. Two days later, the supernatants from hDPSCs with or without YSCH-01 infection were collected and filtered through a 0.45 μm filter. The collected supernatant was stored at − 80 ℃. Tumor cell lines SCC152, HCC1806, U5A, and normal cell line MCF-10A were cultured in 96-well plates at a density of 1 × 10^4^ cells per well, except for SW780, at a density of 6000 cells per well. After 24 h, the medium was removed, and 100 μl of the collected medium (CM2) from hDPSCs with YSCH-01 was added to treat the tumor cells for CCK8 assays. The heat treatment at 56 ℃ for 20 min and ultrafiltration treatment were performed to inactivate or remove the adenovirus, and the tumor cells without supernatant treatment were used as a mock control. Inactivation assay was identified as above protocols, and cell morphology was observed by microscopy after 3 days of culture.

### Statistical analysis

Statistical analysis was performed using GraphPad Prism 8.0 software (GraphPad Software, LaJolla, CA, USA). The measurement data were presented as Mean ± SEM. Comparisons between groups were analyzed using one-way ANOVA or two-way ANOVA. Statistical significance was denoted as: *p < 0.05, ** p < 0.01, ***p < 0.001.

## Results

### Characterization of hDPSCs

Flow cytometry analysis revealed that 99.2% of the cells coexpressed CD44, CD90, and Stro-1, indicating their positive expression of these surface markers (see Appendix Fig. 6A). The colony formation and sphere formation could be observed under a microscope and crystal violet staining (Appendix Fig. 6B–D), indicating its good self-renewal ability. The cell growth viability of hDPSCs was demonstrated for over 7 days (Appendix Fig. 6E). Furthermore, the hDPSCs exhibited the ability to differentiate into neural-like cells, osteoblasts, and adipocytes, as confirmed by immunocytochemistry of neural progenitor markers, such as GAFP and Nestin (Appendix Fig. 6F), positive Alizarin red for osteoblasts mineralization (see Appendix Fig. 6G), and Oil red O staining for adipogenic differentiation (see Appendix Fig. 6H), respectively. These findings indicate that hDPSCs possess the capacity for multi-directional differentiation.

### Optimization on biological fitness of hDPSCs loaded with oncolytic adenovirus

To investigate the biological compatibility between hDPSCs and oncolytic adenovirus, various dosages of Serotype 5 oncolytic adenovirus OncoMul-V2-EGFP were used to infect hDPSCs. The results revealed a dose- and time-dependent increase in exogenous green fluorescent protein expression in the infected hDPSCs. However, the Crystal Violet assay indicated a significant reduction in viable cells when the viral dose exceeded 1500 virus particles (VP) per cell (Fig. 1A). This suggests that a high infectious dose of OncoMul-V2-EGFP had a detrimental effect on hDPSCs viability. In contrast, when hDPSCs were infected with YSCH-01, another oncolytic adenovirus that carried a recombinant type I interferon-like anticancer gene L-IFN, no significant change in cellular viability was observed, even at an infectious dose of up to 2000 VP per cell (Fig. 1B). The CCK8 assay also demonstrated no significant changes in hDPSCs viability within 24 h of infection (Fig. 1C). These results indicate that YSCH-01 exhibits higher safety towards hDPSCs compared to its control virus, OncoMul-V2-EGFP. This safety may be attributed to the limited expression of the serotype 5 adenovirus receptor Coxsackievirus and Adenovirus Receptor (CAR) on hDPSC. Indeed, the qPCR analysis revealed extremely low CAR on hDPSC compared to adenovirus-sensitive cell lines, such as HEK293 and A549 (Appendix Fig. 7A).

Besides, the effect of infection duration on the viral load of dental pulp stem cells was also investigated. First, using the empty virus OncoMul-V2-EGFP to infect hDPSCs at different times, we found that at the same concentration of infection, hDPSCs were infected for 4 h, 7 h, and 12 h. There was no significant difference in the expression of EGFP in hDPSCs at 72 h (Appendix Fig. 7B), which indicated that the infection time did not have a significant effect on the viral load of OncoMul-V2-EGFP/hDPSCs within 12 h. We finally selected 4 h of infection as the construction of YSCH-01/hDPSCs.

Additionally, the objective determination of the number of virus particles loaded into hDPSCs was investigated. The results revealed that the actual intracellular content was approximately 0.77 VP per cell when the initial dose of YSCH-01 was 1000 VP per cell. This indicates that the amount of virus loaded onto hDPSCs was 1298-fold less than the initial infected amount. Consequently, if 2 × 10^6^ cells were injected in vivo, only 1.54 × 10^5^ virus particles would be directly injected (see Table. 1).

**Table 1:** Viral payload of hDPSC after infection with YSCH-01

Experiments	Virus initial infectious dose (VP/cell)	Intracellular viral payload(ifu/cell)	The % index (ifu/VP)	Intracellular VP per cell	Injected cells (YSCH-01/hDPSCs) number in vivo	The amounts of virus delivered by hDPSCs in vivo
EXP 1	1000	5.13 × 10^–3^	0.884	0.58	2 × 10^6^	1.16 × 10^6^
EXP 2	1000	8.43 × 10^–3^	0.878	0.96	2 × 10^6^	1.96 × 10^6^
Average	1000	6.78 × 10^–3^	/	0.77	2 × 10^6^	1.54 × 10^6^

Cellular viral payload of YSCH-01/hDPSCs. The human dental pulp cells were infected with YSCH-01 at a dosage of 1000 VP/cell for 4 h, then washed 3 times with 1 × PBS buffer. After that, the infected hDPSCs were used for detection of the intracellular virus payload by virus titer assay as well as observation of the antitumor efficacy in xenograft mice models through IP injection. Ifu: infectious unit, VP: virus particles.

Furthermore, we assessed the replicative ability of YSCH-01 in hDPSCs and found that YSCH-01 exhibits extremely low replicative capacity in hDPSCs. The viral content within the cells increased only twofold on the 5th day, almost negligible compared to the 2nd day (Appendix Fig. 7C). This very low replicative capacity may also contribute to the limited impact of YSCH-01 on hDPSC vitality.

### Tumor tropism of hDPSC loaded with YSCH-01 in vitro

**Fig. 2:**
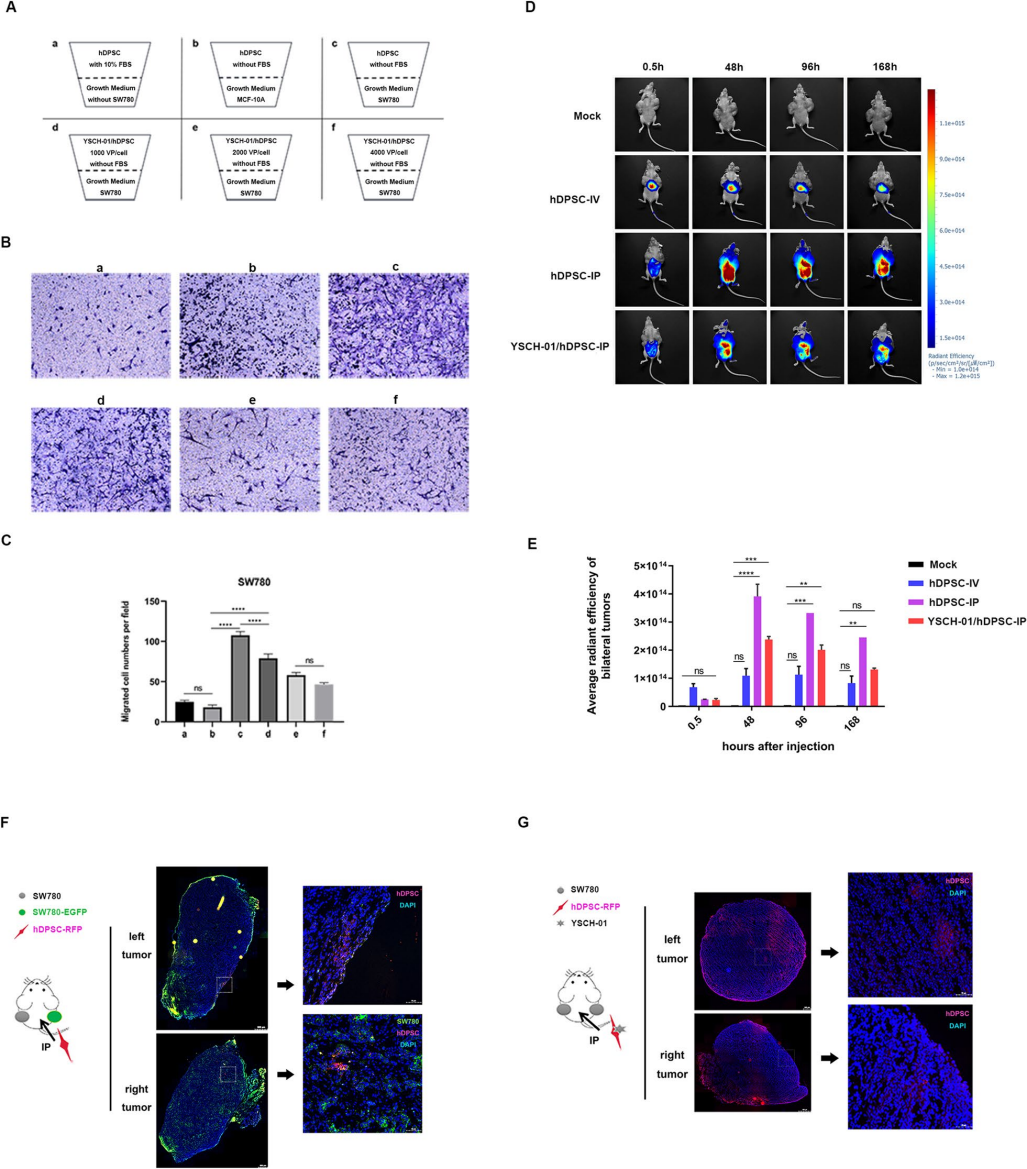
The tumor tropism of hDPSC and YSCH-01/hDPSC. **A** The pattern diagram of designed experimental groups of the transwell assay. **B** Representative images of Crystal violet staining of migrated hDPSCs or YSCH-01/hDPSCs. The magnification is 100**. C** Statistical results of migrated cell numbers are shown in Figure B. **D** Biodistribution of hDPSCs or YSCH-01/hDPSCs in SW780-bearing nude mice model under different systemic administration. **E** Quantitative analysis of fluorescence expression in bilateral tumors in figure D. **F–G** Panoramic scans of the frozen section from tumor tissues treated with hDPSCs-RFP or YSCH-01/hDPSCs-RFP injected intraperitoneally after ten days

As an ideal active cell carrier, it is crucial for hDPSCs not to have their migration ability compromised by the loaded virus during the early stages. We observed that YSCH-01 exhibited minimal toxicity on hDPSCs at dosages up to 1500 VP per cell. However, the tumor tropism of YSCH-01-loaded hDPSCs remained unclear. Transwell assays were performed to investigate this, as shown in Appendix Fig. 7A and Fig. 2A. The result indicated that hDPSC displayed about 3 to fourfold migration ability toward the target SCC152 and SW780 cancer cells (Fig. 2B–c and Appendix Fig. 8B–c). Importantly, even after loading YSCH-01, hDPSCs retained their tumor tropism (Fig. 2B–D and Appendix Fig. 8B–D) while exhibiting no migration towards normal cells like MCF-10A (Fig. 2Bb). Furthermore, it was observed that YSCH-01-loaded hDPSCs maintained their tumor tropism even at an infectious dose of 1000 VP per cell. However, higher infection doses (2000 or 4000 VP per cell) significantly decreased the migration ability of hDPSCs (Fig. 2B-e, B-f). These findings emphasize the importance of selecting an appropriate virus-loading dosage for hDPSCs as a novel cell carrier for oncolytic virotherapy. Therefore, we confirmed that it was reasonable to infect hDPSC at a dose of 1000 VP/cell to construct the YSCH-01/hDPSCs complex.

### Tumor tropism of YSCH-01/hDPSC in xenograft nude mice models

The aforementioned in vitro experiments demonstrated the tumor tropism of both hDPSC and YSCH-01-loaded hDPSCs. However, their behavior in vivo needed to be evaluated. The biodistribution of administrated hDPSCs was initially examined using DiR fluorescence staining and Visque invivo Smart. In the SW780 xenograft model (Fig. 2D), the hDPSCs were detected in both bilateral tumor sites after 48 h of intraperitoneal injection and persisted for at least 168 h. However, no positive signaling was observed in the intravenous group. These findings indicate that the migration of hDPSCs towards tumors in vivo is dependent on the route of injection. Intraperitoneal injection of hDPSCs allowed them to reach the tumor sites, while intravenous administration did not yield the same results. Interestingly, even when loaded with YSCH-01, hDPSCs could only be detected in tumor sites when delivered via intraperitoneal injection (Fig. 2D, E). This suggests that YSCH-01-loaded hDPSCs also exhibit tumor tropism, which aligns with in vitro data.

It is interesting to note how the migration process of IP-injected stem cells targeting tumors occurs (See Appendix Fig. 9A). The results showed that hDPSCs labeled with DIR gradually migrated from the abdominal cavity to the head and neck region starting three hours after self-injection into healthy nude mice. By the 39th hour, there was a significant increase in fluorescence in the head and neck region compared to the 19th-hour time point. However, after injection into bilaterally tumor-bearing nude mice, hDPSCs did not migrate toward the head and neck region after reaching the tumor tissue. Instead, they continued accumulating at the tumor site, continuously increasing fluorescence (Appendix Fig. 9B). This suggests that the migration of hDPSCs is not random dispersion, and in the presence of tumor tissue and chemotactic effects, they continue to migrate specifically into the tumor tissue. Looking at the route via organs from the abdominal injection method, in healthy mice, hDPSCs primarily accumulated in the lungs, spleen, kidneys, and liver tissues, with a higher concentration in the liver and spleen (Appendix Fig. 9C, D). The tumor-bearing model had a higher accumulation in the liver and tumor tissue, followed by the spleen (Appendix Fig. 9C, D). As expected, YSCH-01/hDPSCs showed a migration pattern similar to hDPSCs in healthy and tumor-bearing mice, predominantly accumulating in the liver and tumor tissue (Appendix Fig. 9E–H). Interestingly, YSCH-01/hDPSCs reached their highest fluorescence levels in the tumor at 120 h after entry, followed by a gradual decrease over nine days (Appendix Fig. 9F). This indicates that dental pulp stem cells begin to decrease in cellular activity upon entering the tumor, potentially initiating processes related to apoptosis or cell differentiation.

Additionally, hDPSCs carrying stable RFP expression were constructed and administered to SW780 tumor-bearing mice via intraperitoneal injection. Panoramic scanning of frozen section slices revealed the presence of RFP in tumor tissues in both the hDPSCs-IP or YSCH-01/hDPSCs-IP groups, confirming that hDPSCs reached bilateral tumors, consistent with live imaging results. However, the distribution of hDPSCs within the tumor tissue differed slightly. When hDPSCs were injected alone, they were mainly located in the outer envelope of the tumor. In contrast, when YSCH-01-loaded hDPSCs were injected, the cells were present in the outer envelope and within the tumor parenchyma (Fig. 2F, G).

### Potent anti-tumor effect of YSCH-01/hDPSC by intraperitoneal injection

**Fig. 3:**
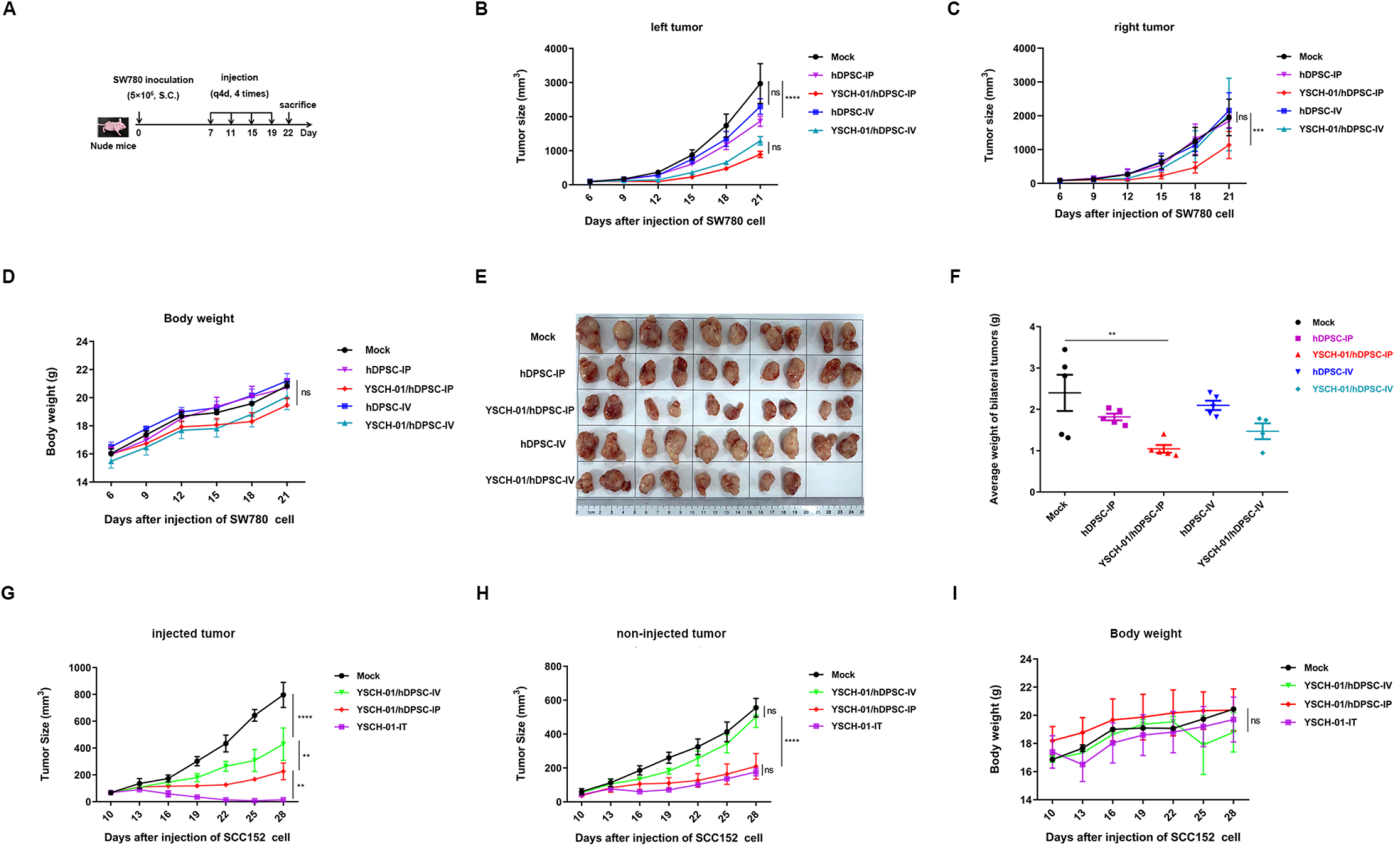
Inhibitory effect of different injection methods of YSCH-01/hDPSC in subcutaneous tumor-bearing mice. **A** The schematic diagram of the operation in nude mice model. **B–C** The growth curves of bilateral tumors in SW780 bearing mice models. **D** The weight curve of mice after different treatments. **E–F** The photos of tumor tissues in each group on the 23rd day and the weight of bilateral tumors in corresponding groups. **G–I** The growth curves of bilateral tumors and the weight curve in SCC152 bearing mice models. IP, IV, and IT refer to intraperitoneal injection, intravenous injection, and intratumoral injection, respectively

Given the confirmed targeting tumor tropism of YSCH-01/hDPSCs, it was hypothesized that intraperitoneal injection of YSCH-01/hDPSCs could effectively inhibit tumor growth. Bilateral subcutaneous SCC152 and SW780 tumor-bearing nude mouse models were further constructed to investigate this. The results demonstrated that intraperitoneal injection of YSCH-01/hDPSCs had a more potent inhibitory effect on bilateral tumors compared to intravenous administration. Interestingly, neither intravenously nor intraperitoneally injected hDPSCs exhibited tumor-inhibitory potential or tumor-promoting effects (Fig. 3A–C, E–H). Furthermore, all treated mice showed no behavioral abnormalities and maintained stable body weight (Fig. 3D, I), indicating the safety of hDPSCs or YSCH-01/hDPSCs injection in vivo.

The IT group exhibits better results than the IP stem cell group for the injected side tumors (Fig. 3G). However, for the non-injected side tumors, the effects of the two methods were comparable, as shown in Fig. 3H. Considering the intracellular viral load mentioned in Table 1, the objectively injected viral load of “YSCH-01/hDPSC” via intraperitoneal injection (2 × 10^5^ ~ 2 × 10^7^ VP) was significantly lower than that of intratumorally purified YSCH-01 virus (2 × 10^9^ VP). hDPSCs carrying the virus being administered at a concentration is approximately 1000 times lower than that in the IT group but achieve outcomes equivalent to the IT group. Also, when intraperitoneal injection of virus 1 × 10^6^ ~ 1 × 10^8^ VP only, this viral quantity was entirely insufficient to inhibit tumor growth, as indicated in Appendix Fig. 8. This suggests that hDPSCs, as a cell carrier, reduce the viral dosage and enable systemic administration of YSCH-01 in vivo, thereby further improving the safety of oncolytic adenovirus administration. Moreover, it is noteworthy that intraperitoneal injection of purified YSCH-01 alone demonstrated no tumor inhibitory effect at a dosage ranging from 2 × 10^5^–2 × 10^7^ VP (Appendix Fig. 10), which is equivalent to the viral dose carried by “YSCH-01/hDPSC.” These findings suggest that, in addition to functioning as a cell carrier for oncolytic adenovirus delivery, hDPSCs may play a crucial role in synergizing with the YSCH-01 virus to enhance the anticancer effect.

### Characterization of hDPSC acting as a cell carrier to deliver oncolytic virus into tumors in vivo

**Fig. 4:**
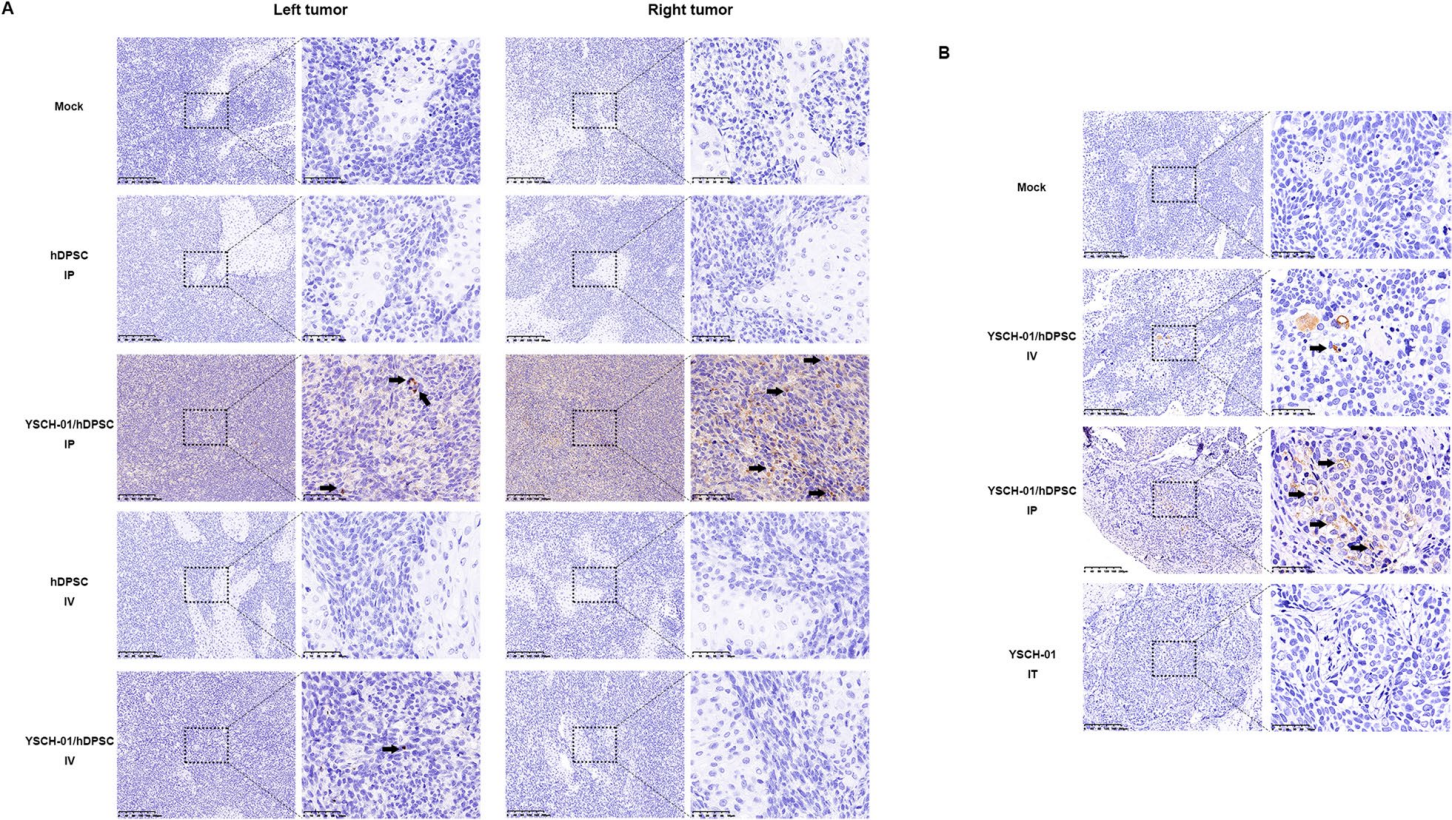
Immunohistochemical images of hexon in tumor tissues under different injection methods. **A** The representative immunohistochemical images of SW780 bilateral tumors. The tumor in each group was treated with hDPSCs -IP, YSCH-01/hDPSCs-IP, hDPSCs-IV, YSCH-01/hDPSCs-IV, respectively. **B** The immunohistochemical results of non-injected tumor tissues in SCC152 bearing mice models. The black arrows in the pictures point to the typical positive hexon protein expression

As demonstrated above, intraperitoneal administration of YSCH-01/hDPSCs exhibited a potent anti-tumor effect. It is also known that hDPSC or YSCH-01/hDPSC can reach tumor sites through systemic administration. However, confirmation of the distribution of the loaded virus was necessary. IHC results revealed that the highest concentration of viral particles was observed in the YSCH-01/hDPSCs-IP group in both the SW780 and SCC152 models (as seen in Fig. 4A, B). This indicates that intraperitoneal injection of YSCH-01/hDPSCs successfully delivers YSCH-01 to bilateral tumor sites, allowing them to infect and lyse tumor cells, thereby exerting a significant suppressive effect.

### Direct tumor cell killing effect of YSCH-01/hDPSC in the co-culture system

**Fig. 5:**
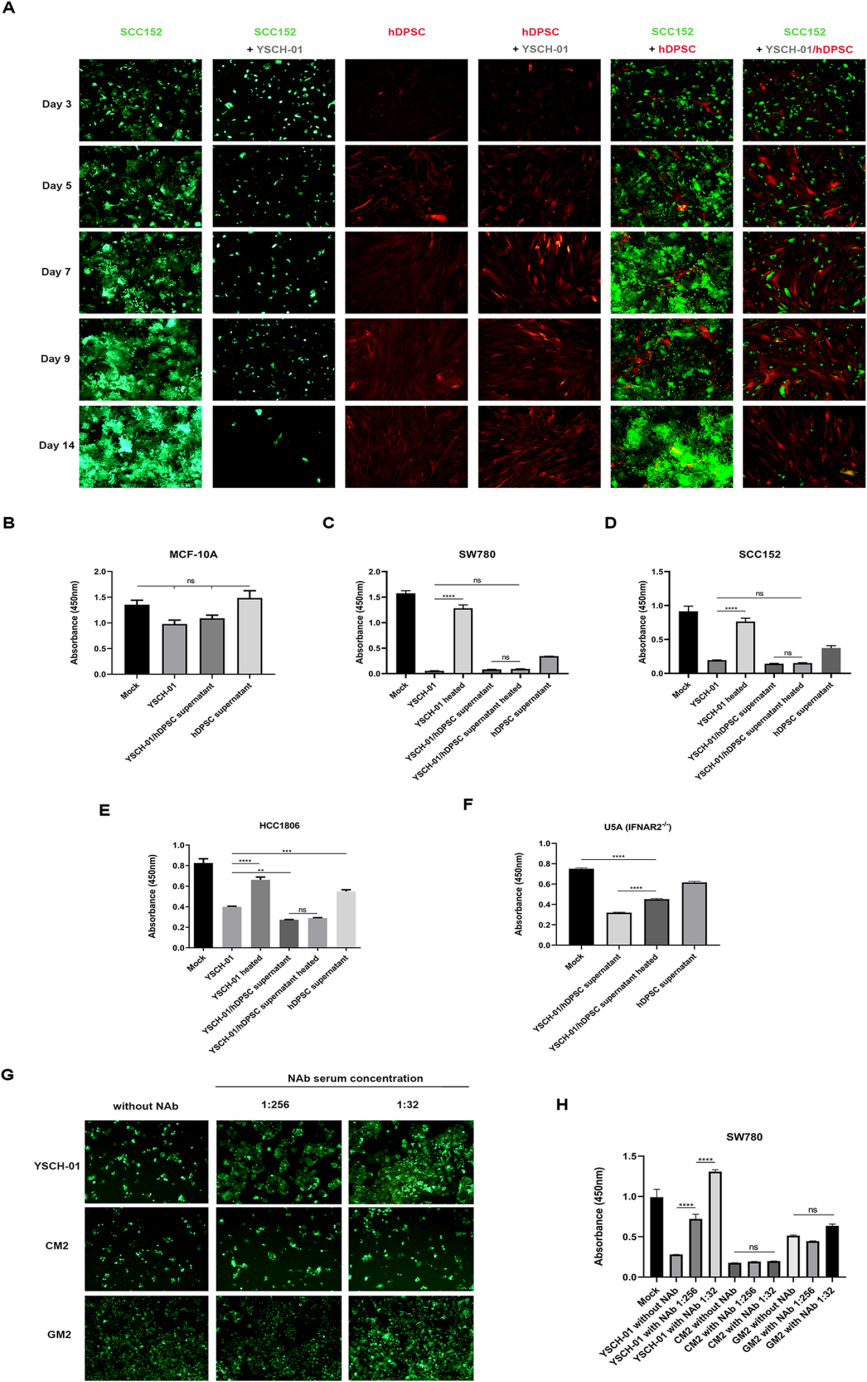
Mechanism of the tumor-killing effect of YSCH-01/hDPSC. **A** Co-culture between tumor cell SCC152 and hDPSCs or YSCH-01/hDPSCs. The magnification is 100. **B–F** The cell viability of normal cell line and tumor cells under the conditioned media from YSCH-01/hDPSCs’ supernatant. Figure B is the result of the normal cell line MCF-10A. Figure C-E show the results of tumor cell line SW780, SCC152, and HCC1806. Figure F shows the results of tumor cell line U5A, which has the IFNaR2 receptor deficiency signaling. **G** The tumor-killing effect of YSCH-01/hDPSCs’ supernatant on SW780-EGFP cells pre-treated with YSCH-01 adenovirus neutralizing antibody (NAb). The magnification is 100. The cells with green fluorescence are the tumor cells. **H** The cell viability of SW780 under the conditioned medium, including NAb. The GM2 was the 48-h supernatant from the growth medium of hDPSCs and the CM2 indicated the 48-h supernatant from YSCH-01/hDPSCs

In order to investigate the mechanism of YSCH-01/hDPSC, a co-culture system was designed to observe the dynamic behavior of two cell types over time. The fluorescence of GFP in groups without YSCH-01 treatment (SCC152-EGFP, SW780-EGFP, hDPSC-RFP co-cultured with SCC152-EGFP, hDPSC-RFP co-cultured with SW780-EGFP) was significantly elevated (Fig. 5A and Appendix Fig. 11). This suggests that tumor cells exhibited a superior growth tendency compared to hDPSCs. However, the EGFP fluorescence decreased dramatically in the YSCH-01/treated groups or YSCH-01/hDPSC-treated groups. Additionally, numerous tumor cells were observed to detach and eventually disappear after several days. Conversely, the proliferation of hDPSCs was noticeably enhanced in the YSCH-01/hDPSC-treated group. In contrast, the viability of hDPSCs in the hDPSC-treated groups progressively weakened over time. These findings collectively demonstrate that tumor cells exhibited a competitive growth advantage over hDPSCs when co-cultured without YSCH-01. However, when hDPSC carried YSCH-01, the co-culture microenvironment underwent a significant change. YSCH-01/hDPSC disrupted the defense mechanism of tumor cells, leading to their subsequent death and ultimately securing their survival in this competitive environment. Interestingly, when YSCH-01/hDPSCs and SW780 cells were co-cultured with different effector-target ratios ranging from 1:1 to 1:5, there was no discernible difference in the tumor inhibitory effect (Appendix Fig. 12).

### The YSCH-01/hDPSC supernatant exerted tumor-specific cell inhibitory effect in both an active oncolytic virus and an exogenous protein-independent manner

In addition to the direct killing effect of YSCH-01/hDPSC in the co-culture system, other mechanisms need to be explored. One potential mechanism could be the effect of supernatant. Firstly, it was observed that the supernatant of YSCH-01/hDPSC had no toxicity on normal breast fibroblast cell line MCF-10A, indicating its safety to normal cell lines (Fig. 5B). However, a significant inhibitory effect on cell growth (Fig. 5C–E) and cell morphology (data not shown) was observed in the human cancer cell lines SCC152, SW780, and HCC1806 after treatment with the supernatant from YSCH-01/hDPSCs. On the other hand, the secretome of hDPSCs showed a moderate inhibitory effect without evident changes in cell morphology. This suggests that the active virus released by YSCH-01/hDPSC into the supernatant was responsible for killing tumor cells. When YSCH-01 was heated or ultrafiltrated, there was nearly no active oncolytic virus exerting anti-tumor effect (Appendix Fig. 13). However, surprisingly, even when the supernatant was heated to 56 ℃ for 20 min to inactivate the released virus, the three types of tumor cells exhibited a sharp loss in viability and showed no difference with YSCH-01/hDPSC’s supernatant without heat treatment (Fig. 5C–E). These results indicate that the supernatant of YSCH-01/hDPSC either exerts its tumor-specific cell growth inhibitory effect in a YSCH-01-independent manner.

Furthermore, considering the presence of inserted L-IFN expression in YSCH-01 and its contribution to the anti-tumor effect, an IFNAR2^−/−^ fibrosarcoma cell line U5A, which essentially requires the IFNAR2 receptor for molecular function, was used. Similarly, a significant inhibitory effect on U5A cells was observed after treatment with heated YSCH-01/hDPSC supernatant (Fig. 5F). This suggests that the supernatant possesses a tumor-specific killing effect independent of both oncolytic virus and L-IFN. It is important to further investigate whether other anti-tumor cytokines, miRNAs, and exosomes are present in the secretion of YSCH-01/hDPSCs.

## Discussion

MSCs have shown potential as cell delivery vehicles for oncolytic viruses, offering a new approach to cancer therapy. However, there is currently no report demonstrating whether human dental pulp-derived MSCs possess the same properties as a cell carrier for cancer therapy. Recent studies have highlighted the unique advantages of hDPSCs. Firstly, hDPSCs are abundant in wisdom teeth and can be easily obtained compared to MSCs derived from other tissues. Secondly, hDPSCs exhibit a higher proliferative capacity than bone marrow-derived mesenchymal stem cells (BMSCs) [26, 27]. Additionally, hDPSCs secrete a greater quantity of growth factors, such as NGF and BDNF, compared to BMSCs and adipose-derived mesenchymal stem cells (ADSCs) [28]. These characteristics suggest that hDPSCs may have broader clinical applications compared to MSCs derived from other tissues. Therefore, we propose that dental pulp stem cells could serve as a novel cell carrier for delivering oncolytic viruses in cancer therapy. As depicted in Figs. 1 and 3, this study demonstrates that hDPSCs have the potential to carry a sufficient number of oncolytic adenovirus YSCH-01 particles and retain their ability to migrate to tumor cells. Notably, both nude mice (Fig. 3) and humanized mice models (data not shown) exhibited potent anticancer effects. The selected oncolytic virus, YSCH-01, is a serotype 5 adenovirus modified in the E1A-CR2 region, driven by a tumor-specific survivin promoter, and inserted with a multifunctional anticancer L-IFN gene. Our data suggests an infection dosage of 1000 VP per cell with a 4-h infection duration is suitable for maintaining host cell viability and motility (Fig. 2, and Appendix Fig. 7). However, the intracellular viral particle count in Table. 1 indicates a relatively low level, which may be attributed to the low expression of the CAR receptor on hDPSCs. It is worth noting that there are alternative mechanisms for recombinant adenoviruses to enter host cells, such as αvβ5 integrin [29], HSPG, VCAM-1, MHC-Iα2 [30], MARCO [31], and others. Moreover, determining the optimal infection duration is essential. Figure 1A demonstrates a positive correlation between adenovirus-armed exogenous EGFP expression level in hDPSCs and the infection time.

Although the viral loading in hDPSCs was relatively low at approximately 0.77 VP per hDPSC, our findings revealed a significant and robust anti-tumor effect upon intraperitoneal injection of YSCH-01/hDPSC (Fig. 3). This effect was comparable to that observed with intratumoral injection of purified YSCH-01. Notably, to achieve similar anti-tumor efficacy, the intraperitoneal injection of YSCH-01/dDPSC at a dosage of 1.54 × 10^6^ VP in vivo was only about one-thousandth of the dosage (2 × 10^9^ VP) required for intratumoral purified YSCH-01 injection (Appendix Table 1). Furthermore, we verified that intratumoral injection of low doses of YSCH-01 (ranging from 2 × 10^5^ to 2 × 10^7^ VP), equivalent to the viral dosage carried by YSCH-01/hDPSCs, had no impact on tumor growth compared to the mock group. These results indicated that low-dose intratumoral treatment alone did not exhibit any anti-tumor effect (Appendix Fig. 8). Therefore, this suggests that the strategy of utilizing hDPSCs as a carrier for oncolytic adenovirus enables the achievement of anti-tumor efficacy with low viral dosages, thus improving the safety profile of systemic adenovirus administration.

Interestingly, this study demonstrated the role of hDPSCs as essential drug delivery vehicles and revealed their synergistic effect with YSCH-01 in enhancing the anti-tumor response. Importantly, it was confirmed that the supernatant derived from YSCH-01/hDPSC exhibited a tumor-specific inhibitory effect on multiple tumor cells while showing no toxicity toward normal cells. Notably, this effect was independent of the active YSCH-01 adenovirus and secreted L-IFN protein (Fig. 5F).

Regarding the detailed mechanism underlying the anticancer effect of YSCH-01/hDPSC supernatant, it is postulated that, in addition to exogenous L-IFN expression and viral cell lysis, the gene expression profile of YSCH-01-infected hDPSCs may undergo significant changes, leading to the secretion of unknown bioactive substances into the cellular supernatant. These substances could include cytokines, proteins, nucleic acids, uncoated vesicles, or other anti-tumor exosomes. Previous reports have highlighted that DPSC-conditioned medium collected under normoxic conditions is enriched with anti-inflammatory and regenerative factors, thereby stimulating further investigation into its therapeutic applications [32]. However, to date, there have been few reports on the in vivo anti-tumor effects of hDPSC supernatant or hDPSCs loaded with antitumor drugs. Therefore, future research will focus on identifying and characterizing the active anti-tumor secretome present in the supernatant derived from YSCH-01/hDPSCs.

It has been previously reported that hDPSCs exhibit significant migration towards tumor cells in in vitro assays. However, the tumor tropism of hDPSCs could not be confirmed in vivo following intravenous, intraperitoneal, or peritumoral injection under the tested experimental conditions [16]. In contrast, our results confirmed the tumor tropism of hDPSCs and demonstrated the anti-cancer efficacy of YSCH-01/hDPSC in vivo. Additionally, we observed distinct characteristics between the two injection methods in the nude mice model. Figures 2D, E and 4A, B indicated that the number of hDPSCs reaching the tumor site was significantly lower following intravenous injection than intraperitoneal injection, with a more pronounced antitumor efficacy observed in the peritoneal group. These differences may be attributed to the physical barrier of intravenous injection, the unique microenvironment of the abdominal cavity, and potentially influenced by the activity of hDPSCs or the specific animal model selected [33–35].

Regarding the evaluation of antitumor efficacy in xenograft nude mice models, it is important to note that both the oncolytic adenovirus and hDPSCs used in this study are of human origin, which may not fully replicate the regulation of human anti-tumor immunity due to the immunodeficiency of nude mice lacking T cells and non-humanized immune cells like NK cells. To address this limitation, we conducted a pilot experiment using a newly developed humanized mice model, where we found that intravenous injection of YSCH-01/hDPSCs also exhibited a potent anti-tumor effect, with an approximately 95% growth inhibitory rate in an SCC152 xenograft model (data not shown). Therefore, further investigation is warranted to elucidate the anti-tumor mechanism under different injection methods, mainly studying the YSCH-01/hDPSCs-induced anti-tumor immunity in humanized models.

Additionally, it is crucial to thoroughly evaluate stem cells' fate in xenograft models. Figure 2F, G shows that hDPSCs could persist in tumor sites for at least 9 days following intraperitoneal injection. However, whether they eventually undergo cell death or differentiate is unknown and needs further exploration. Some studies have reported that MSCs gradually die in vivo, while others have shown that MSCs can differentiate into various cell types at the tumor site [36, 37]. For instance, it has been demonstrated that BMSCs tend to undergo osteogenic differentiation in metastatic lung tumors and lipogenic differentiation in subcutaneous tumors [38]. Moreover, the differentiation ability of stem cells in vivo can be influenced by the number of cells used, which may be related to the negative feedback regulation of the organism [39]. Morever, it has been observed that after being exposed to a specific tumor microenvironment, MSCs can differentiate into tumor-associated fibroblasts (TAF)-like cells or cancer-associated fibroblasts (CAFs) through signaling pathways such as TGF- β/Smad/Notch [37] and PGFR-β [40], promoting tumor growth.

Subcutaneous cell tumors can be rapidly established to create inhibitory tumor models, providing a convenient way to monitor tumor growth and the anti-cancer effects of drugs. However, compared to orthotopic tumor models, the microenvironment in subcutaneous tumor models differs, which may lead to varying chemotactic effects for drug-loaded cellular carriers with migratory properties. Consequently, differences in anti-cancer efficacy might arise. Considering cell-based approaches for cancer therapy, for indications like periodontitis, subcutaneous injection of stem cells seems suitable. However, when it comes to using virus-carrying stem cells for anti-cancer purposes, we believe that direct virus injection for cancer treatment is sufficient. Nonetheless, the idea of injecting virus-carrying cells directly for cancer treatment is worth discussing. It could potentially enhance the broader distribution and infiltration of the virus within solid tumor tissues. Leveraging the migratory properties of cells could enhance viral dissemination, thus achieving a more effective anti-cancer outcome.

Our study has highlighted further areas to explore; however, unanswered questions remain. In our future studies, we intend to investigate additional receptors that can potentially enhance YSCH-01's entry into human dental pulp stem cells (hDPSCs). Furthermore, we plan to investigate the underlying mechanisms responsible for the synergistic effects observed between YSCH-01 and hDPSCs. We will also aim to identify the essential components within the secretome of YSCH-01-loaded hDPSCs that play a significant role in exerting anti-cancer effects. Additionally, we will explore the specific immune cells involved in the anti-cancer immune response. Lastly, we plan to examine the fate of hDPSCs after they have exerted their anti-tumor effects in vivo.

## Conclusion

In summary, this is the first study reporting that the use of hDPSCs as a carrier for delivering oncolytic adenovirus YSCH-01 demonstrates a potent anti-tumor effect, involving both the direct viral lysis and the secretion of specific anti-tumor bioactive substances by the cells. YSCH-01-loaded hDPSCs are capable of exhibiting tumor tropism and effectively targeting tumor sites. The supernatant derived from YSCH-01/hDPSCs has broad tumor-specific cell inhibitory effects, independent of both the oncolytic virus and its inserted anticancer gene L-IFN. This study highlights the clinical potential of hDPSCs as a novel cell carrier in the field of oncolytic virus-based cancer therapy.

## Figures and Tables

**Fig. 6:**
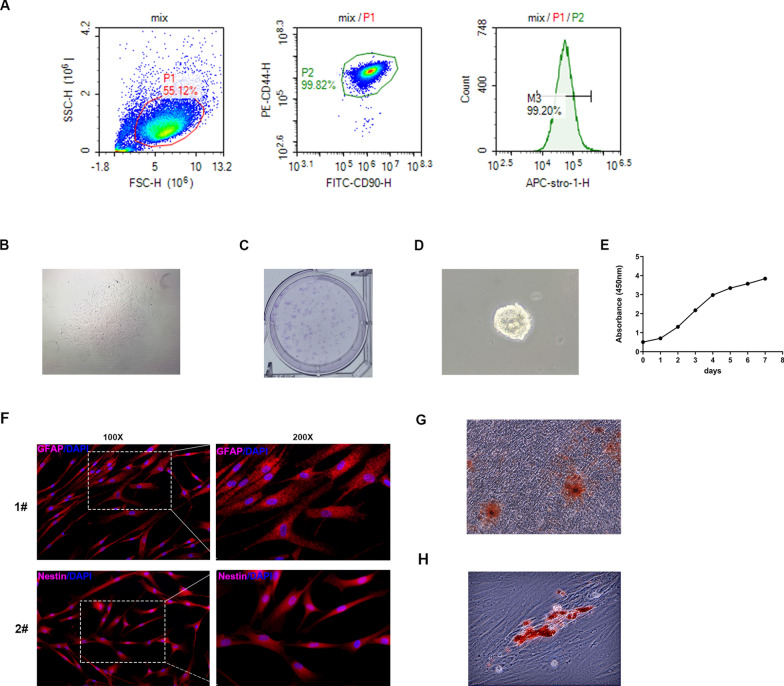
Identification of hDPSC. **A** Surface biomarkers STRO-1, CD90, and CD44 were detected to identify hDPSCs followed by flow cytometry. **B** The representative microscopic morphology of colony formation. The magnification is 40. **C** The representative crystal violet staining of colony formation. **D** The representative microscopic observation of sphere formation. The magnification is 200. **E** The growth curve of selected hDPSCs. **F** Representative immunofluorescence images of neural-related biomarker GFAP and Nestin on the 8-day differentiated cells from hDPSCs in neural induction medium. 1# and 2# represented two independent fields. The magnification of the left side is 200, and the right side is 400. **G** Representative image of Alizarin red staining. The magnification of the microscope is 200. **H** Representative image of Oil Red O staining. The magnification is 400

**Fig. 7:**
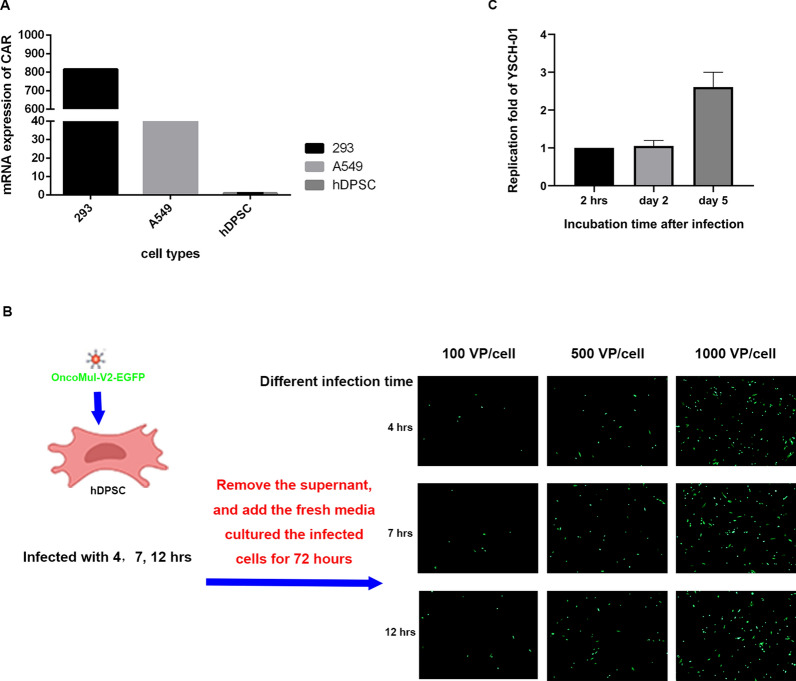
The replication of oncolytic virus in hDPSC. **A** The relative mRNA expression level of adenovirus receptor CAR in three different cell types. **B** Diagram and EGFP expression level in hDPSCs infected with OncoMul-V2-EGFP for different infection times and the magnification is 40. **C** Detection of viral replication in hDPSCs after hDPSCs infected with YSCH-01 for 2 h, 2 days, and 5 days, respectively

**Fig. 8:**
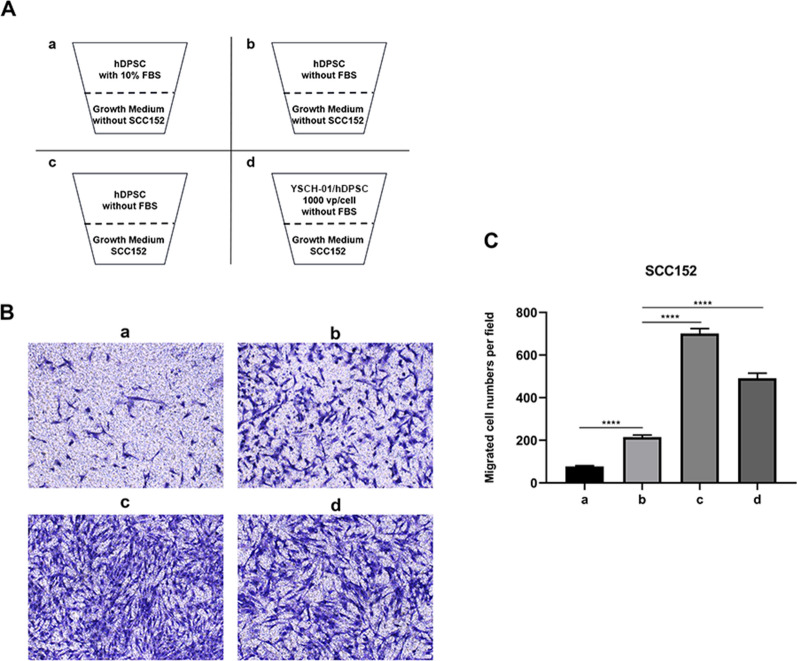
Migration of hDPSCs and YSCH-01/hDPSCs towards the SCC152 cell line. **A** The pattern diagram of designed experimental groups of the transwell assay. **B** Representative images of Crystal violet staining of migrated hDPSCs or YSCH-01/hDPSCs. Each group in this experiment was replicated three times. The magnification of the microscope is 100. **C** Statistical results of migrated cell numbers are shown in Figure B

**Fig. 9:**
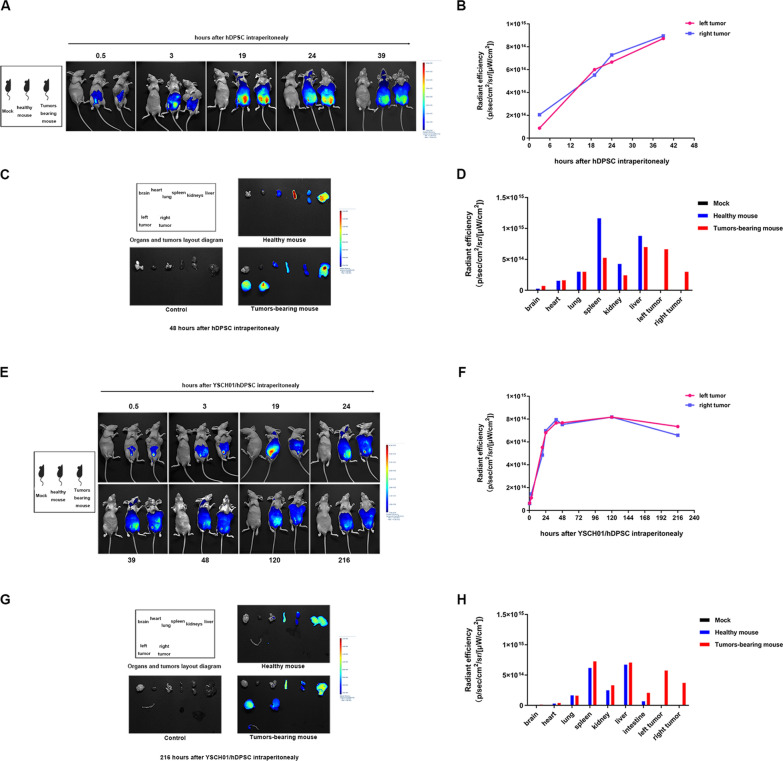
The distribution and lifespan of hDPSC and YSCH-01/hDPSC in vivo. **A** Biodistribution of hDPSCs in healthy and SW780-bearing mouse model. **B** Quantitative analysis of fluorescence expression in bilateral tumors in figure A. **C** The fluorescence of separated organs and tumors. **D** Quantitative analysis of fluorescence expression in bilateral tumors in figure C. **E** Biodistribution of YSCH-01/hDPSCs in healthy and SW780-bearing mouse model. **F** Quantitative analysis of fluorescence expression in bilateral tumors in figure E. **G** The fluorescence of separated organs and tumors. **H** Quantitative analysis of fluorescence expression in bilateral tumors in Figure G

**Fig. 10:**
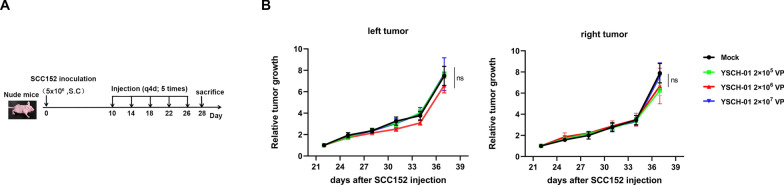
The intraperitoneal injection of low-dosage YSCH-01 in the SCC152-bearing nude mice model demonstrated no tumor inhibitory effect. **A** Schedule of low-dosage YSCH-01’s injection. **B** The growth curves of bilateral tumors showed no difference between low dosage YSCH-01 treated groups and mock group

**Fig. 11:**
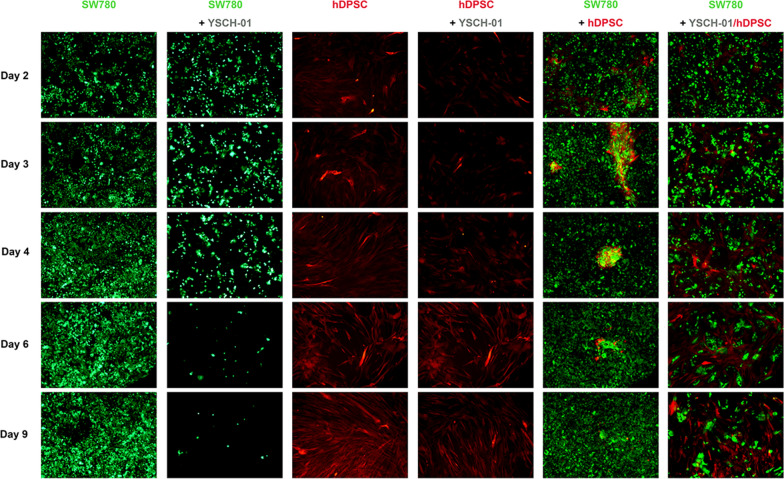
Co-culture between SW780 tumor cell line and hDPSCs or YSCH-01/hDPSCs in vitro. The SW780-EGFP tumor cells were mostly killed when co-cultured with YSCH-01/hDPSCs-RFP, while not with hDPSCs-RFP only. Both tumor cells and hDPSCs or YSCH-01/hDPSCs were co-cultured at the same starting cell numbers in a 6-well plate for indicated days

**Fig. 12:**
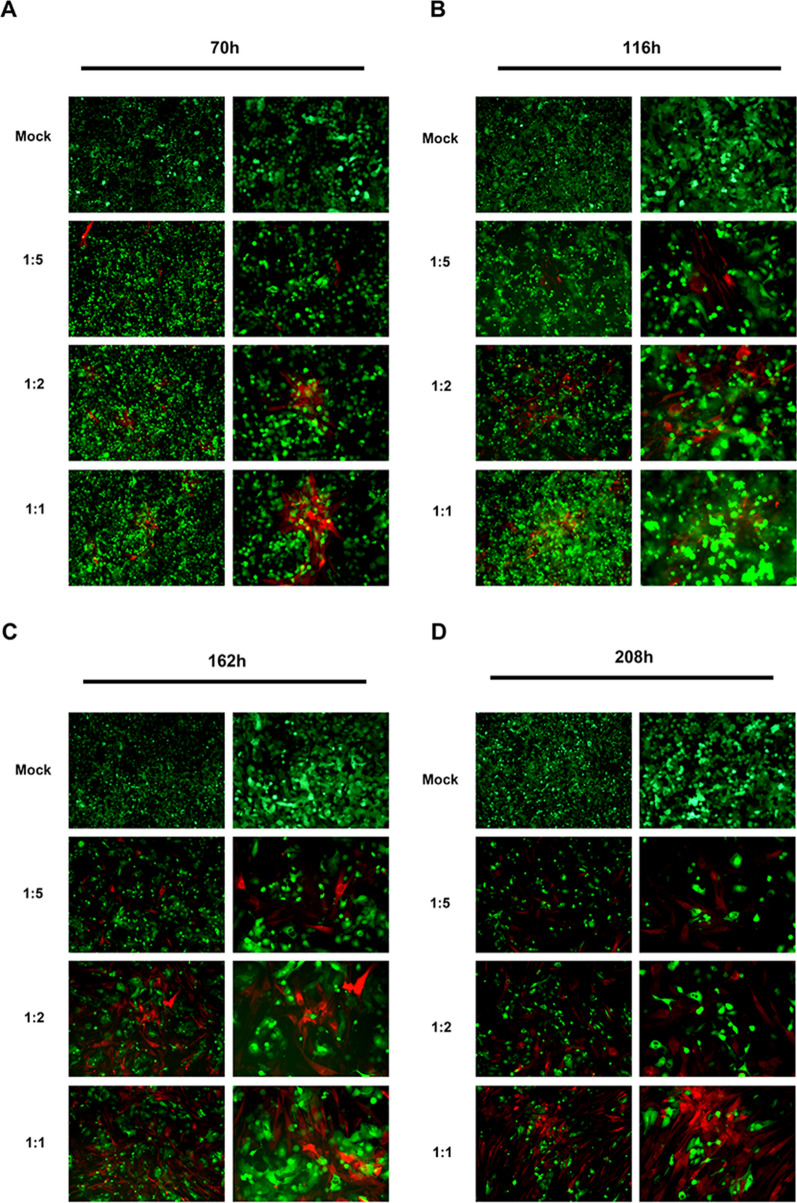
Different effect-target ratios showed no difference in the killing effect on tumor cells in vitro. **A-D** Except mock, YSCH-01/hDPSCs exerted a similar inhibitory effect on tumor cells among different effect target ratio groups by time. The microscope magnification of the left columns in Figure A-D is 40 and the microscope magnification of the right column in each figure is 100

**Fig. 13:**
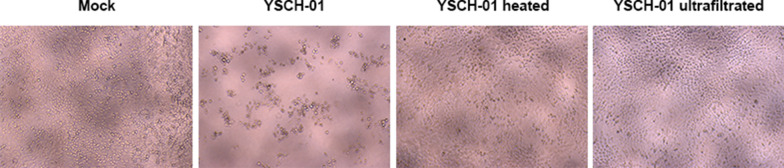
The identification of inactivated virus. Mock: Without any treatment. YSCH-01: Cultured with YSCH-01 dilution. YSCH-01 heated: Cultured with YSCH-01 dilution treated by 56℃ water bath for 20 min. YSCH-01 ultrafiltrated: Cultured with YSCH-01 dilution treated by 100 kDa ultrafiltration tube centrifugal

## Data Availability

The data and materials which supported the findings in this research are available upon reasonable request to the corresponding author.

## Appendix

**See **Figs. 6, 7, 8, 9, 10, 11, 12, 13

## Abbreviations

hPDSCs: Human dental pulp stem cells
MSC_S_: Mesenchymal stem cells
HSV: Herpes simplex virus
YSCH-01/hDPSCs: hDPSCs armed with oncolytic adenovirus YSCH-01
VP: Virus particles
CAR: Coxsackievirus and adenovirus receptor
BMSCs: Bone marrow-derived mesenchymal stem cells
ADSCs: Adipose-derived mesenchymal stem cells
